# Sudden Death of a Teenager Caused by *Actinomyces israelii*: A Case Report

**Published:** 2018-09

**Authors:** Carmen Corina RADU, Andreea CAMARASAN, Cristina Maria PODILA, Dan PERJU-DUMBRAVA

**Affiliations:** 1. University of Oradea, Faculty of Medicine and Pharmacy, Oradea, Romania; 2. Legal Medicine Bihor County Service, Oradea, Romania; 3. Iuliu Hatieganu University of Medicine and Pharmacy, Cluj-Napoca, Romania

**Keywords:** *Actinomyces israelii*, Sudden death, Medico-legal autopsy

## Abstract

Actinomycosis is a rare bacterial infection, caused by a group of Gram-positive bacteria which form the normal flora of oral cavity, gastrointestinal tract, and female genital tract. We present a rare case met in 2016 at Legal Medicine Bihor County Service, Romania of a 14 yr old boy in infection with *Actinomyces israelii* was able to produce sudden death. Infection with *A. israelii* was diagnosed with the help of histological exam, after medico-legal autopsy. Most probably, *A. israelii* was aspirated from the level of the oropharynx, it arrived into the lungs, and using the haematological way it spread into the heart, causing sudden death.

## Introduction

The actinomycetes are a group of bacteria found in putrefied vegetables and in the soil, but we found in the literature cases described in humans and animals too. Actinomycetes include anaerobic bacteria (facultative anaerobic bacteria) which belong to *Actinomyces* genus and aerobic bacteria classified into two groups depending by the presence or absence of mycolic acids in the cell wall-actinomycetes with mycolic acids and without mycolic acid (1).

Actinomycosis is a bacterial infection, usually uncommon, caused by a group of anaerobic Gram-positive bacteria which form the normal flora of oral cavity, gastrointestinal tract and female genital tract (2, 3). Usually, the infection appears at people who present risk factors like local tissue damage caused by trauma, recent surgery (splenectomy) or irradiation, alcoholism, use of steroids, HIV, diabetes, leukemia with chemotherapy, renal or/and lung transplants (4). It can also appear in women with intrauterine devices leading to pelvic actinomycosis (4).

There are 30 species of Actinomyces, but only eight of them can be met by human beings (2). *A. israelii* is the most common type of Actinomyces which cause human infection (5). It usually affects the orocervicofacial region, thoracic region, abdominal-pelvic region and central nervous system (2). Its pathway depends on its form, therefore, thoracic form of actinomycosis usually appears after introduction in the body of the bacteria through esophageal perforation, by direct spread from an actinomycotic process of the neck or abdomen, or via hematogenous spread from a distant lesion. If there are mucosal disruptions, the bacteria lead to infections in the cervicofacial region. Sometimes the infected material from the oropharynx can be aspirated so that the *A. Israelii* can spread in the human organism, it can arrive into the lungs, gives nonspecific complications and in the end, it can lead to death (2).

## Case report

We present a male case of a 14 yr old 2016 met at Legal Medicine Bihor County Service, Romania who bought an energy drink and while he was consuming it, he was feeling sick and felt down on the ground. He was resuscitated for about 1 hour, but unfortunately, he was declared dead. After we discussed with his family, he felt ill for two weeks, accusing chest pain, drowsiness, fatigue, productive cough and low-grade fever treated withtylenol and aspirin. He was at attending physician the day before his death and he was given tylenol and aspirin because he was diagnosed as having flu. No complementary examination like complete blood count or imaging exams (computed tomography) was made.

We followed the human subjects’ procedure, established by our institution. The research was conducted with the rules of good conduct in scientific research. The identity of the participant in the research is confidential when the results of this study are published. Informed consent was taken from the relatives of the patient.

### Autopsy findings

External exam of the corpse did not reveal any violent lesions neither pathological.

Examination of the oral cavity showed normal dentition, but the gums corresponding to the teeth 1, 1, 1, 2.1, 3, 2.1, 2.2 were red and swollen, specific for gingivitis. There was no visible sign of illness at the level of head and neck so we could exclude the orocervicofacial form of this disease.

**Fig. 1:**
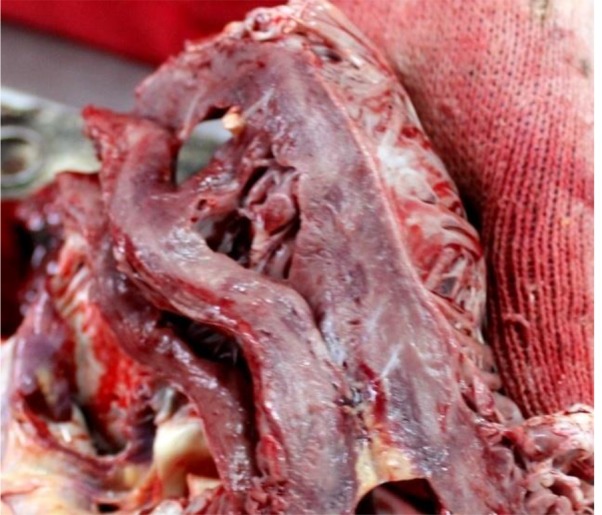
The aspect of the heart-section (macroscopic): myocarditis

**Fig. 2:**
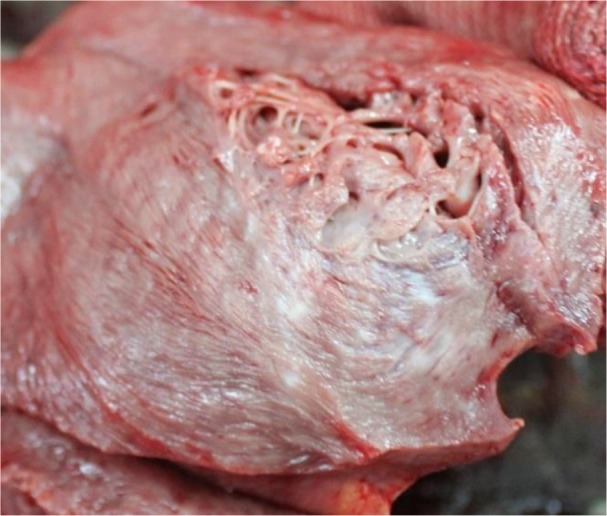
The aspect of the heart-section (macroscopic): cardiosclerosis

Macroscopic the lungs were described as pneumonia, emphysema and pulmonary edema; the entire myocardium looked like myocarditis (Fig. 1) and on the posterior wall of the left ventricle was an area with cardiosclerosis (Fig. 2) which made us think that it could be a scar from a myocardial infarction.

### Microscopic exam

**Fig. 3:**
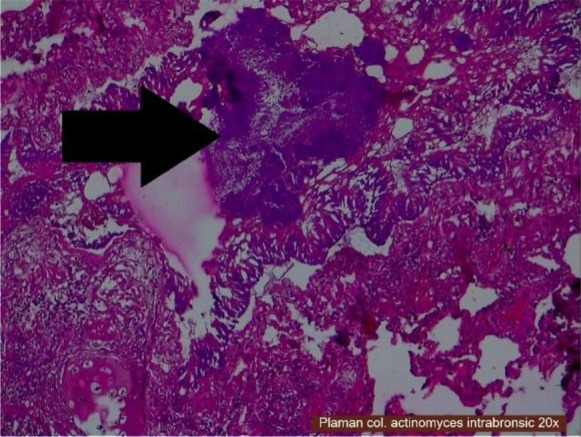
Lung, HE staining, 20X-Actinomyces Israelii inside the bronchi

**Fig. 4:**
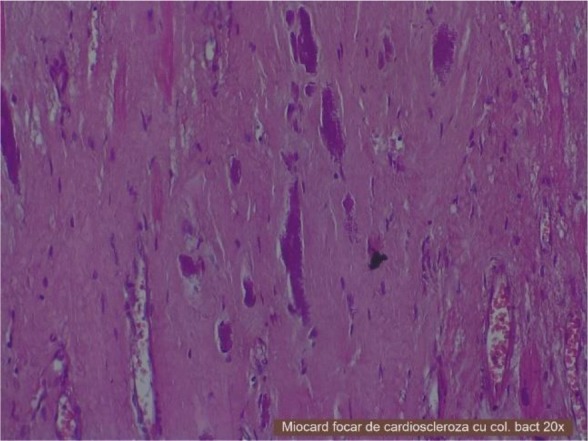
Heart, HE staining, 20X-cardiosclerosis with bacterial colonies

Inside the bronchi from the lungs inflammatory granulomas with *A. israelii* (not stained periodic acid Schiff-PAS negatively) were visible (Fig. 3). In myocardium bacterial colonies, microabscesses, diffuse cardiosclerosis, myocytes with necrosis and polymorphonuclear cells were visible (Fig. 4). Therefore microscopic exam revealed acute myocardial infarction, heart failure, myocardosclerosis, chronic myocarditis and into the lungs infection with *A. israelii*.

Corroborating the clinical features, autopsy findings and complementary examinations it was established that this sudden death was caused by an acute myocardial infarction as a result of chronic myocarditis and pulmonary pneumonia with *A. israelii*.

## Discussion

Actinomycosis is a rare disease caused by a group of anaerobic Gram-positive bacteria normally found in the oral cavity, gastrointestinal tract and urogenital tract (6, 7). *Actinomyces* are related to Nocardia species and in the past, they were considered fungi because of their branching filaments, but today they are classified as bacteria (6). *A. israelii* is met as a normal part of the normal anaerobic flora of the throat, tonsillar crypts, and mouth, also it can be found in the membranes lining the intestines and vagina (8). Usually, the infection appears at people who present risk factors like local tissue damage caused by trauma, recent surgery (splenectomy) or irradiation, alcoholism, use of steroids, HIV, diabetes, leukemia with chemotherapy, renal or/and lung transplants (4). It can also appear in women with intrauterine devices leading to pelvic actinomycosis (4). Apparently, there is no relationship between race, occupation or season (9). According to the anatomic site of infection, actinomycosis can be classified inorocervicofacial, thoracic, abdominal, pelvic, central nervous system, musculoskeletal and disseminated (4). The most common form of actinomycosis is cervicofacial, followed by thoracic form which appears in 15% to 30% of actinomycosis cases, more frequent at males (male: female ratio 3:1) in young to middle-aged adults (aged 20–50 yr) except pelvic form which is more frequent at women (10, 11). Actinomycosis also can appear in children and teenagers, in a study made of 85 cases of actinomycosis, 27% were in persons under 20 yr old, 7% being children under 10 yr old (9).

The orocervicofacial infection occurs when there is a breach in the mucosal lining so that the bacteria can penetrate local structure (dental extraction or trauma to the mouth, poor dental hygiene, periodontal disease, neoplasms or osteonecrosis of the jaw from radiation treatments) (4, 8).

### Thoracic form

Humans are the natural reservoir for this bacteria, but when the gastrointestinal secretion or infected material from the oropharynx is aspirated, even if bacteria are not normally virulent, it can enter into the body (1, 2, 7, 8, 11). The patient with thoracic actinomycosis usually has nonspecific symptoms and signs (2).

The heart is affected in less than 2% with *A. israelii* (7). Microscopic aspects for myocardium are typical, with necrotizing of abscess, forming masses of mycelia bodies and sulfur granules.(5). These findings are highly suggestive of the diagnosis but are not specific, as they can been countered in other pathogenic conditions (nocardiosis) (7).

In most cases actinomycosis mimics pneumonitis and it can cause productive cough, chest pain, weight loss, dyspnea and fever (2). When the myocardium is affected although cardiac symptoms are usually absent, sometimes the patient may present chest pain (5).

Abdomen and pelvic actinomycosis: For abdominal form of actinomycosis, usually there is a history of recent intestines surgery or perforations by foreign body and it involves the ileocecal region. A pelvic form of this disease commonly appears in women as an ascending infection from the uterus, being associated with intrauterine devices (12, 13).

Central nervous system form is rare and cerebral abscesses are met; they appear on the computer tomography as a ring lesion with a thick wall which can be irregular or nodular (13).

Musculoskeletal form occurs because of the spreading of the infection from the infected tissues near the muscles and bones, but sometimes can be associated with traumatic lesions like fractures (mandibular fractures) or it can spread via haematological way (13).

After infection with *A. israelii*, the immune system of the infected host simulates an inflammatory response as suppurativegranulomatous and fibrotic reaction and then it spreads and invades surrounding tissues and organs. In the end, the infection products draining sinus tracts, full with damaged tissue. From this site, the infection can disseminate through blood circulation to distant organs (11).

For the diagnosis of actinomycosis two conditions must be respected: positive cultures, or sulfur granules on the histopathological exam. Histological examination of the heart tissue reveals an outer zone of granulation tissue and a central zone of necrosis containing many granules that represent microcolonies of actinomyces (14).

Even if microscopic exam of the heart was not specific for infection with *A. israelii*, the microscopic diagnoses as chronic bacterial myocarditis lead us to the assumption that it is the same bacteria from the lungs which spread via haematological way and arrived into the heart.

There are no recent estimates of the disease prevalence; a study reported 28 cases with actinomycosis. In most cases (92%) the diagnosis of actinomycosis was not suspected on admission. Nine patients had abdominal/pelvic form, 5 orocervicofacial and 5 cardiothoracic. The median age was 52 yr, and 50% males. From all 5 patients with cardiothoracic form of this disease, one patient presented pneumonia with nonspecific interstitial infiltrate and another one had mitral valve endocarditis through hematogenous spread from an infected implantable port (15). At forensic autopsy, we found that the lungs were macroscopically specific for pneumonia and at histological exam, made from the lung sample, was discovered that the boy was infected with *A. israelii*. Only one patient from all 28 included in a study, presented the same form, like ours, of thoracic actinomycosis, and another one had endocarditis through hematogenous spread from an infected implantable port. Maybe in our case, the trigger for myocarditis was pneumonia with Actinomyces through haematogenous way.

The study Pulmonary actinomycosis during the first decade of 21st century: cases of 94 patients reported that in 10 yr, in 13 hospitals from Korea were registered 94 (66 males and 28 females) cases with pulmonary actinomycosis. Pulmonary actinomycosis occurred frequently in middle to old-aged people, men-age being 57.7 yr old, the majority of them presenting comorbidities. They presented symptoms like cough, hemoptysis, and sputum production, dyspnea, fever, and chest pain (16). We observed that the symptoms were similar to our patient, involving cough, low-grade fever and chest pain, productive cough but no hemoptysis. Moreover, in our research, patient had no associated comorbidities, his constitution was athletic, he had no cardiovascular risk factors, nothing documented capable to produce sudden death (17). The consumption of energy drinks is not cited in literature as being a risk factor.

Corroborating all the aspects, clinical, macroscopic and microscopic findings we established that the most probable explanation for this sudden death was infection with *A. israelii* which from the level of oropharynx entered into the lungs and also into blood, and on haematological way spread into the heart, gave myocarditis with acute myocardial infarction and finally led to death.

Because the disease is very rare, there is little known about effective preventive measures. However, the prevention should include maintenance of good oral hygiene and adequate regular dental care (19, 20). In addition, the use of appropriate antibiotic prophylaxis (penicillin) when the mouth or gastrointestinal tract is penetrated can lower the risk of this kind of infections (21).

## Conclusion

Infection with *A. israelii* is very rarely seen in medical practice, these bacteria forming the normal flora of oral cavity, gastrointestinal tract, and female genital tract. We need to highlight the exceptional form in which the bacteria manifested: therefore, it gave cardio-respiratory symptoms leading to thoracic type of this disease. Most probably the bacteria was aspirated from the oropharynx so it arrived into the lungs, and using the haematological way it spread into the heart, causing sudden death. The dissemination of the bacteria into the heart led to chronic myocarditis and on pathological myocytes the heart developed an acute myocardial infarction, causing sudden death.

## Ethical considerations

Ethical issues (Including plagiarism, informed consent, misconduct, data fabrication and/or falsification, double publication and/or submission, redundancy, etc.) have been completely observed by the authors.
